# Is It Helpful to Believe That Efforts Will Lead to Positive Outcomes? Two Cross-Lagged Panel Investigations among Adolescents and Young Adults

**DOI:** 10.3390/ijerph17207585

**Published:** 2020-10-19

**Authors:** Jacky C. K. Ng, Vince W. T. Cheung, Helen S. M. Wong, Sherry M. Y. Leung, Victor C. Y. Lau

**Affiliations:** 1 Department of Counselling and Psychology, Hong Kong Shue Yan University, Hong Kong, China; 2 Department of Applied Social Sciences, Hong Kong Polytechnic University, Hong Kong, China; wing-tung-vince.cheung@polyu.edu.hk; 3 Division of Business and Hospitality Management, Hong Kong Polytechnic University, Hong Kong, China; helen.wong@cpce-polyu.edu.hk (H.S.M.W.); sherry.leung@cpce-polyu.edu.hk (S.M.Y.L.); 4 Department of Social Work, Hong Kong Baptist University, Hong Kong, China; victorlau_27@yahoo.com.hk

**Keywords:** reward for application, life satisfaction, worldviews, self-views, self-esteem, young adults, adolescents

## Abstract

Over the past few decades, the role of self-views in life satisfaction has been extensively investigated. Recently, growing attention has been directed to the question of whether an optimistic worldview, termed “reward for application”, helps boost life satisfaction. Conceptually, the association between reward for application and life satisfaction can be paradoxical. Due to various methodological and theoretical shortfalls, previous investigations were unable to draw a robust conclusion on this association. To address these shortfalls, two cross-lagged panel studies were conducted with different time lags. Over and above the potential confounds of self-views (namely, self-esteem and self-rated personality traits), reward for application had a positive effect on lagged life satisfaction among both adolescents and young adults, while the reverse effect was not found. Moreover, we found support for the multiplicative effect between worldviews and self-views, in which the positive effect of reward for application on life satisfaction was attenuated by high self-esteem.

## 1. Introduction

The study of what predicts life satisfaction is an interesting one, but what makes some people happier than others? Myers and Diener [1] reviewed many studies to identify certain “happy traits” such as self-esteem and the personality trait of extraversion. Twenty years on, and still this line of investigation continues and remains influential [2], with researchers finding that personality traits and self-esteem have consistent and substantial associations with life satisfaction [3,4]. DeNeve and Cooper [5] meta-analyzed 244 independent studies and found small to moderate correlations between big five personality traits and life satisfaction (*r*s = 0.14 to 0.24). Diener and Diener [6] meta-analyzed data from approximately 12,600 participants across 31 nations and found a moderately strong association between self-esteem and life satisfaction (*r* = 0.47). Self-esteem reflects a positive evaluation towards the self, while self-rated personality traits reflect one’s perception of the self and the evaluation of one’s own attributes. Previous research conceptualized these two psychological variables as self-views, which represent beliefs and perceptions about oneself [7,8].

As well as focusing on one’s perceptions about the self (self-views), research has also examined perceptions about the world (worldviews) and its effects on life satisfaction [9]. One worldview, termed “reward for application”, is especially relevant to life satisfaction because it has implications for another two happy traits identified by Myers and Diener [1]: optimism and personal control. Reward for application highlights an optimistic belief and reflects a sense of agentic control [10]. Therefore, in the present research, we investigated the effect of reward for application on life satisfaction.

While life satisfaction has been extensively studied over the years, many of the studies suffer from methodological and theoretical shortfalls. Methodologically, most previous studies on worldviews and life satisfaction employed cross-sectional designs among young adults, restricting the robustness and generalizability of the findings. Theoretically, previous studies have predominantly focused on the additive effects of worldviews and self-views on life satisfaction, overlooking the possibility of multiplicative effects between worldviews and self-views. To address these research gaps, we therefore conducted two longitudinal studies among both young adults and adolescents, testing both additive and multiplicative effects of reward for application on life satisfaction.

### 1.1. Worldviews: Differentiation from Self-Views

Worldviews refer to a set of social beliefs that highlights one’s perceptions about the world in which one functions [11]. This research operationalized worldviews as a set of “social axioms” [12], which were defined as “generalized beliefs about people, social groups, social institutions, the physical environment, or the spiritual world as well as about categories of events and phenomena in the social world” [13] (p. 198). In their examination of the factorial validity of social axioms, Leung and Bond [14] identified a pan-cultural five-factor model that applied across 40 cultures. The five-factor structure of social axioms at the individual level was also validated in other multicultural studies [15,16]. The five factors are: (a) *social cynicism*, which indicates a pessimistic view of human nature and social institutions, (b) *reward for application*, which refers to the optimistic belief that persistent efforts can overcome challenges and difficulties, and bring positive outcomes, (c) *fate control*, which highlights the belief that life events are predetermined and influenced by impersonal, external forces, (d) *social complexity*, the belief that there are multiple solutions to problems, and (e) *religiosity*, which describes a belief in spiritual forces and the positive social effects of religious practices. Conceptually, social axioms are captured by the pure belief items that assert the relationship between two external entities or concepts, which are exogenous to the self [12]. Empirically, Lai, Bond and Hui [17] demonstrated small-to-moderate correlations between social axioms and self-esteem (*r*s = 0.01 to 0.39, with an average of 0.16). Chen, Fok, Bond and Matsumoto [18] also demonstrated small-to-moderate correlations between social axioms and self-rated big five personality traits (*r*s = 0.02 to 0.42, with an average of 0.15).

Among the five factors of social axioms, social cynicism and reward for application are especially relevant to life satisfaction. Social cynicism highlights a pessimistic worldview while reward for application reflects an optimistic worldview. They showed consistent associations with life satisfaction across previous studies [9]. The longitudinal association between social cynicism and indicators of psychological health has been investigated in previous research. For instance, Lai and colleagues [17] found that social cynicism was negatively associated with life satisfaction one year later. In a three-month cross-lagged panel study, Ng [19] observed a negative lagged effect of social cynicism on life satisfaction and happiness. Leung, Ip and Leung [20] conducted a one-year cross-lagged panel study among working adults and found that social cynicism had a negative lagged effect on job satisfaction. However, unlike social cynicism, only limited research has examined the association between reward for application and life satisfaction with a longitudinal design. Therefore, the present research attempts to fill this gap.

### 1.2. An Optimistic Worldview: Reward for Application

Reward for application refers to a belief that the exertion of effort will lead to positive outcomes [12]. People high in reward for application believe that they can resolve challenges and achieve positive outcomes through hard work and thoughtful investment of resources [14]. Thus, reward for application can be regarded as an optimistic worldview. It is noteworthy that while reward for application may imply optimism, it is not equivalent to optimism. Optimism reflects a belief in the high likelihood of having positive outcomes in the future, while reward for application implies a belief that human agency is empowered by knowledge, effort, and careful planning [10]. Therefore, the optimism implicit in reward for application is deeply rooted in the effort exerted, rather than coming from nowhere. Empirical findings supported this notion. Hui and Bond [10] only observed a modest correlation between reward for application and optimism (*r* = 0.24). Bernardo and Nalipay [21] found that reward for application was associated with the loci-of-hope dimensions that highlight human agency and was not associated with the dimension that focuses on supernatural and spiritual forces.

As a worldview highlighting one’s agentic control over actions and outcomes, reward for application was positively associated with the exertion of effort [22]. Furthermore, reward for application motivated people to take an active role in goal-attainment, Nalipay, Bernardo and Mordeno [23] revealed that reward for application encouraged people to proactively engage in adaptive cognitive processing and was associated with great post-traumatic growth after a natural disaster. To resolve interpersonal conflicts, Bond, Leung, Au, Tong, and Chemonges-Nielson [24] found that people high in reward for application adopted active coping styles such as compromising, accommodating, and collaborative strategies. Overall, reward for application has been described as a “good force” to promote positive psychological outcomes, such as better intercultural adjustment [25] and dyadic adjustment [26].

#### 1.2.1. Reward for Application and Life Satisfaction: A Paradoxical Relationship

Conceptually, the relationship between reward for application and life satisfaction can be paradoxical. One might intuitively expect a positive association between reward for application and life satisfaction—as a force driving positive outcomes (e.g., successful intercultural and dyadic adjustment), reward for application might be expected to promote life satisfaction. Centered on a belief that goals are achievable through effort, reward for application was found to link positively to the exertion of effort and positive attitude to effort [22]. By endeavoring to achieve, people high in reward for application may accomplish more, which in turn brings greater levels of satisfaction with their lives.

Nevertheless, it is possible that reward for application may fail to promote life satisfaction. Although it intuitively makes sense that effortful behaviors can lead to accomplishments and life satisfaction, it is not always true. Research has shown that reward for application was associated with learning morale but showed no positive effect on academic accomplishment [22]. Similarly, Leung, Chen and Lam [27] observed that reward for application was positively associated with academic ambition but showed no association with academic accomplishment. As a whole, it seems that reward for application can motivate people to exert effort, but does not help them accomplish more, restricting its ability to promote life satisfaction.

In fact, because effortful behaviors do not guarantee success, reward for application may even diminish life satisfaction. Singelis, Hubbard, Her, and An [28] found that people high in reward for application tended to continue striving for success even after a failure. Reward for application may therefore increase the imbalance between effort spent and reward received [29], which has been shown to be detrimental to life satisfaction [30].

Briefly put, the relationship between reward for application and life satisfaction can be paradoxical. A positive association makes intuitive sense, whereas it is also possible to hypothesize an absence of positive association or even a negative association. Previous research has attempted to shed light on this association, but the robustness of such findings was largely limited by methodological shortfalls.

#### 1.2.2. The Methodological Shortfalls in Previous Studies

The relationship between reward for application and life satisfaction has been widely examined in the literature. For instance, Chen, Cheung, Bond and Leung [31] found a positive correlation between reward for application and life satisfaction among young adults in China (*r* = 0.14). Safdar and colleagues [25] found a positive correlation among young adults in Iran (*r* = 0.35). Recently, Chen and colleagues [9] investigated different samples in Hong Kong and also observed positive correlations among children (*r* = 0.36), adolescents (*r* = 0.25), and young adults (*r* = 0.27). Although the majority of the previous findings revealed a positive relationship, Hui and Bond [10] found a null relationship between reward for application and life satisfaction among young adults in Hong Kong [17].

Based on the aforementioned studies, it is tempting to conclude a positive relationship between reward for application and life satisfaction. Nonetheless, it is noteworthy that all of these studies are cross-sectional studies, and this methodological flaw might restrict a robust conclusion. Since the measures of reward for application and life satisfaction are based on self-report, measuring these variables at the same time point (i.e., a cross-sectional design) can be subject to the threat of common method variance [32]. Thus, it is possible that the observed positive correlation might result from the common method factor, suggesting that reward for application and life satisfaction might have a null or negative relationship. When measures are based on self-report, one remedy to lower the threat of common method variance is “to create a temporal separation by introducing a time lag between the measurements of the predictor and criterion variables” [33] (p. 887). Additionally, a cross-sectional design does not allow an investigation into temporal directions. We cannot be sure whether the positive correlation reflects the effect of reward for application on life satisfaction or, in fact, the reverse.

Another methodological shortfall in previous research regards the control variables. To predict life satisfaction, the control of self-views is especially important because they have been shown to have great impact on life satisfaction [2]. For instance, when examining the association between reward for application and life satisfaction, some previous researchers did not rule out the confounding effects by controlling self-views [25,34], while others did control self-views [9,10]. In these studies, self-rated personality traits and self-esteem were regarded as two important self-views and were controlled in the analysis.

Consequently, methodological flaws in previous studies have limited our ability to conclude a robust relationship between reward for application and life satisfaction. To overcome these shortfalls, we conducted two cross-lagged panel studies to deal with the common method variance [33], examine the temporal directions between reward for application and life satisfaction [35], and control the confounding effects of self-views (e.g., self-rated personality traits and self-esteem). Two samples of young adults and adolescents were also recruited to strengthen the generalizability of the findings.

### 1.3. When Worldviews Meet Self-Views: An Interplay Relationship

In the literature, it is common to examine the additive effects of worldviews and self-views, such as whether self-views showed incremental predictions beyond worldviews or whether worldviews had incremental predictability over and above self-views [7]. Theoretically, other than their additive effects, the interplay between worldviews and self-views could predict various psychological outcomes. Person–situation interaction has long been explored [36,37]. How one views of oneself in terms of characteristic attributes may interact with the social environment in predicting psychological outcomes. However, social environment is not necessarily objective, and people do not see the world and environment as they are. Instead, people subjectively see them through a filter [38]. These subjective perceptions of the social environment are reflected by one’s worldviews. As a result, built upon the person–situation interaction, it is possible that worldviews and self-views may interact with each other in predicting psychological outcomes.

In the literature, all previous studies on reward for application and life satisfaction focused on the additive effects, leaving the possible multiplicative effects between reward for application and various self-views unexplored. For instance, it is possible that the big five personality trait of conscientiousness interacts with reward for application to affect life satisfaction. The positive effect of reward for application on life satisfaction may be strengthened among conscientious people who are responsible, well-organized and careful because these people are more likely to be rewarded for their effortful and persistent behaviors [39,40]. Moreover, it is possible for self-esteem to interact with reward for application in predicting life satisfaction. Leung and Bond [13] proposed that reward for application is especially related to one’s psychological well-being under negative or difficult situations because the effect of being persistent can be more noticeable in difficult times. Therefore, the positive effect of reward for application may be more evident among people with a negative perception of self-worth (i.e., low self-esteem).

### 1.4. The Present Research

To summarize, our objectives were threefold.

First, the relationship between reward for application and life satisfaction can be conceptually paradoxical, such that a positive or even a negative association can be expected. Although previous researchers have attempted to study this association, their cross-sectional findings were unable to provide a robust conclusion due to various methodological flaws, namely common method variance, a failure to capture temporal dynamics, and a failure to control self-views. Hence, to overcome these methodological shortfalls, we took a longitudinal approach and conducted two cross-lagged panel studies in this research (*n* = 409).

Second, most of the previous studies on worldviews and psychological well-being were conducted among young adults, restricting the generalizability of the findings. In the present research, the two cross-lagged panel studies were conducted on both young adults (study 1) and adolescents (study 2). To further strengthen the generalizability of findings, we varied the time frame across the two studies. A relatively shorter time lag (three months) was used in study 1 while a relatively longer time lag (one year) was adopted in study 2.

Third, we aimed at going beyond the additive effects of worldviews and self-views on life satisfaction found in previous studies by investigating the multiplicative effects between worldviews and self-views. Given that an interplay relationship can be theoretically possible, we examined the interaction effect between reward for application and two sets of self-views (namely, self-rated personality traits in study 1 and self-esteem in study 2) on life satisfaction.

## 2. Study 1

### 2.1. Method

#### 2.1.1. Participants and Procedure

Two hundred and seven young adults were recruited from a college in Hong Kong (119 females; *M_age_* = 19.49, *SD* = 0.91). These participants were recruited through distributing leaflets and posting announcements in classes at the beginning of a semester. They were also recruited on the basis that they are able to read and comprehend Chinese as the Chinese version of questionnaire was used in this research.

Upon recruitment, the details of the present research were provided to the participants. After informed consent was obtained and confidentiality was ensured, they were asked to complete an online questionnaire at home. The questionnaire consisted of two key constructs, namely reward for application and life satisfaction. They also reported demographic information (namely, age and gender) and self-rated big five personality traits. To investigate the temporal dynamics between reward for application and life satisfaction, each participant’s levels of reward for application and life satisfaction were followed up approximately three months later.

All subjects gave their informed consent for inclusion before they participated in the study. The study was conducted in accordance with the Declaration of Helsinki 1975, and the protocol was approved by the Human Subjects Ethics Sub-Committee of the Hong Kong Polytechnic University (HSEARS20131217002).

#### 2.1.2. Measures

Reward for Application. The 8 item subscale of reward for application was extracted from the Social Axioms Survey (SAS-2) [16] to measure the belief that efforts invested in human resources will lead to positive outcomes. Responses on each belief statement were anchored on a 5 point Likert scale ranging from 1 (*strongly disbelieve*) to 5 (*strongly believe*). A sample item is “One will succeed if he/she really tries” (α = 0.85 and 0.85 for time 1 and time 2, respectively).

Life Satisfaction. The 5 item Satisfaction with Life Scale [41] was used to measure an overall evaluation of one’s life. Responses on each item were anchored on 7 point scales ranging from 1 (*strongly disagree*) to 7 (*strongly agree*). A sample item is “In most ways my life is close to my ideal” (α = 0.89 and 0.89 for time 1 and time 2, respectively).

Personality Traits. The 20 item Mini-International Personality Item Pool [42] was used to measure how participants viewed themselves in terms of five personality factors. Responses were anchored on a 5 point Likert scale ranging from 1 (*does not describe me at all*) to 5 (*describes me very well*) (α = 0.71, 0.68, 0.69, 0.60, and 0.48 for neuroticism, extraversion, openness to experience, agreeableness, and conscientiousness, respectively).

#### 2.1.3. Data Analysis Plan

As a first step to examine the temporal dynamics between reward for application and life satisfaction, we employed confirmatory factor analysis and tested the factorial invariance of reward for application and life satisfaction between time 1 and time 2 to ensure the temporal equivalence in measurements. In this step, parceling was used with two to three items being randomly combined into three parcels [43].

Second, to examine the temporal dynamics between reward for application and life satisfaction, a cross-lagged panel model was established with structural equation modeling. In this model, the lagged effect of reward for application at time 1 on life satisfaction at time 2 was tested with the control of the autoregressive effect from life satisfaction at time 1. Similarly, the lagged effect of life satisfaction at time 1 on reward for application at time 2 was also tested with the control of the autoregressive effect from reward for application at time 1.

Assessment of model fit was based on multiple criteria, including absolute misfit and incremental fit indices. A model with root-mean-square errors of approximation (RMSEA) < 0.08, standardized root mean squared residual (SRMR) < 0.08, non-normed fit index (NNFI) > 0.90, and comparative fit index (CFI) > 0.90 was considered as having acceptable fit to the data [44].

### 2.2. Results

Descriptive statistics and bivariate correlations are summarized in Table 1. A moderate level of autoregressive correlation between constructs at time 1 and time 2 was observed, *r* = 0.55, *p* < 0.001 (reward for application), *r* = 0.51, *p* < 0.001 (life satisfaction). These results reveal that there was around 70% to 74% of unexplained variance in the two constructs across time, making temporal prediction of changes feasible.

#### 2.2.1. The Temporal Dynamics between Reward for Application and Life Satisfaction

First, before we examined the temporal predictions, we tested the factorial invariance of reward for application and life satisfaction over time to ensure temporal equivalence. To do this, a confirmatory factor analytic model was established with three parcels loaded on a latent factor of reward for application and three parcels loaded on a latent factor of life satisfaction in each time point, yielding four latent factors in total. Then, the confirmatory factor analytic model with factor loadings fixed as equal across time (i.e., a constrained model) was compared with the confirmatory factor analytic model with freely estimated factor loadings (i.e., an unconstrained model). Results reveal that the model fit in the constrained model did not drop significantly, Δχ^2^ (4) = 0.53, *p* = 0.970, indicating that the factor loadings were equivalent across time. Additionally, the confirmatory factor analytic model with these constraints fitted the data well, χ^2^ (52) = 100.93, *p* < 0.001, CFI = 0.96, NNFI = 0.95, SRMR = 0.05, RMSEA = 0.07. All factor loadings were statistically significant ranging from 0.66 to 0.96, *ps* < 0.001, with an average of 0.79.

**Figure 1:**
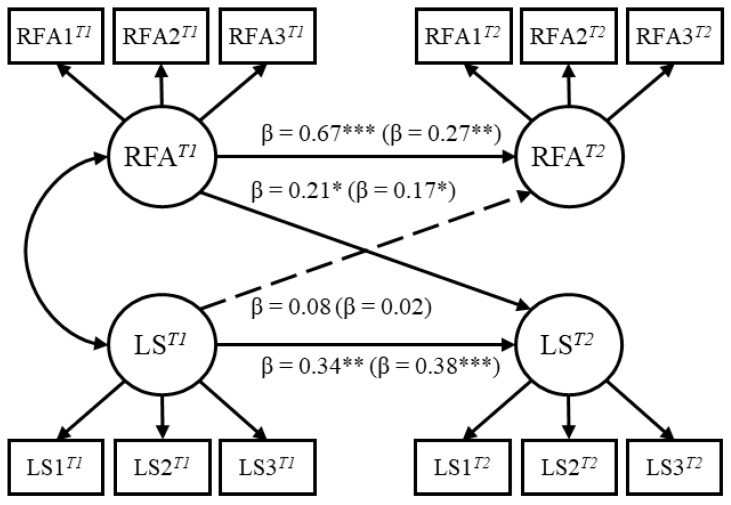
The structural equation model examining the temporal dynamics between reward for application and life satisfaction in study 1 (outside parentheses) and study 2 (inside parentheses). RFA = reward for application; LS = life satisfaction. All the coefficients are standardized, and the solid lines indicate statistical significance. RFA1*^T1^* refers to the first parcel of reward for application at time 1; LS1*^T1^* refers to the first parcel of life satisfaction at time 1. * *p* < 0.05, ** *p* < 0.01, *** *p* < 0.001.

Second, to examine the temporal predictions, a cross-lagged panel model was fitted to the data (see Figure 1). To rule out possible confounding effects, self-rated personality traits and demographic information of age and gender were controlled in the model. Overall, the panel model fitted the data well, χ^2^ (108) = 166.09, *p* < 0.001, CFI = 0.96, NNFI = 0.93, SRMR = 0.04, RMSEA = 0.05. For the structural relations, life satisfaction at time 1 positively predicted life satisfaction at time 2, *b* = 0.40, β = 0.34, *p* = 0.001, indicating a significant autoregressive effect. After controlling the autoregressive effect of life satisfaction at time 1 and the effects of personality traits, reward for application at time 1 still significantly predicted life satisfaction at time 2, *b* = 0.63, β = 0.21, *p* = 0.014. On the other hand, reward for application at time 1 positively predicted reward for application at time 2, *b* = 0.77, β = 0.67, *p* < 0.001, indicating a significant autoregressive effect. However, with the control of reward for application at time 1 and personality traits, life satisfaction at time 1 failed to predict reward for application at time 2, *b* = 0.04, β = 0.08, *p* = 0.430.

#### 2.2.2. The Interplay between Reward for Application and Personality Traits

To examine the interaction effects between reward for application and self-rated personality traits, we tested whether the lagged effect of reward for application on life satisfaction would be moderated by the self-rated personality traits. Latent moderation analysis was performed using the latent moderated structural equations (LMS) approach [45]. This approach can handle the violation of the distributional assumption in product term by representing the multivariate normal distribution of the joint indicator as a finite mixture of normal distributions. Built upon the above cross-lagged panel model, we tested the latent moderation effects from five personality traits on the link between reward for application at time 1 and life satisfaction at time 2. Results indicate that none of the moderation effects were significant, *b*s = −0.45 to 0.15, *ps* > 0.380. Further, none of the moderation effects from the five personality traits on the link between life satisfaction at time 1 and reward for application at time 2 were observed, *b*s = −0.06 to 0.09, *ps* > 0.095.

## 3. Study 2

### 3.1. Method

#### 3.1.1. Participants and Procedure

Two hundred and two adolescents were recruited from five different grades in a secondary school in Hong Kong (equivalent to grades 7 to 11 in the American school system) with ages ranging from 11 to 17 (89 females; *M_age_* = 13.11, *SD* = 1.29). These participants were recruited through distributing leaflets and posting announcements in classes at the beginning of a semester. They were also recruited on the basis that they are able to read and comprehend Chinese as the Chinese version of questionnaire was used in this research.

Upon recruitment, the details of the present research were provided to the participants and a paper-pencil questionnaire package was given to them. After informed consent was obtained and confidentiality was ensured from both the participants and their parents, they were asked to complete a paper-pencil questionnaire at home. The questionnaire consisted of two key constructs, namely reward for application and life satisfaction. They also reported demographic information (namely, age and gender) and self-esteem. Approximately one year later, each participant’s levels of reward for application and life satisfaction were followed up.

#### 3.1.2. Measures

Reward for Application. The 9 item subscale of reward for application was extracted from the short version of the Social Axioms Survey (SAS-1) [14] to measure the belief that efforts invested in human resources will lead to positive outcomes (α = 0.81 and 0.88 for time 1 and time 2, respectively).

Life Satisfaction. The 5 item Satisfaction with Life Scale [41] was used to measure the overall evaluation of one’s life (α = 0.82 and 0.93 for time 1 and time 2, respectively).

Self-Esteem. The 10 item Rosenberg Self-Esteem Scale [46] was used to measure how participants viewed themselves in terms of self-worth. The items were anchored on 4 point scales ranging from 1 (*strongly disagree*) to 4 (*strongly agree*). A sample item is “I have a number of good qualities” (α = 0.80).

#### 3.1.3. Data Analysis Plan

The data-analytic procedure was the same as that described in study 1.

### 3.2. Results

Descriptive statistics and bivariate correlations are summarized in Table 1. The autoregressive correlations between constructs at time 1 and time 2 were found to be significant, *r* = 0.24, *p* < 0.001 (reward for application), *r* = 0.44, *p* < 0.001 (life satisfaction), indicating at least 81% of unexplained variance in the constructs.

#### 3.2.1. The Temporal Dynamics between Reward for Application and Life Satisfaction

First, a confirmatory factor analytic model (i.e., three parcels loaded on a latent factor of reward for application and three parcels loaded on a latent factor of life satisfaction in each time point, yielding four latent factors in total) with constraints on factor loadings was compared with the unconstrained model. Results reveal that the model fit in the constrained model did not drop significantly, Δχ^2^ (4) = 0.38, *p* = 0.984, indicating that the factor loadings were equivalent across time. Furthermore, the constrained model fitted the data well, χ^2^ (52) = 109.24, *p* < 0.001, CFI = 0.96, NNFI = 0.94, SRMR = 0.05, RMSEA = 0.07. All factor loadings were statistically significant, ranging from 0.56 to 0.97, *ps* < 0.001, with an average of 0.79. Second, a cross-lagged panel model was fitted to the data with the control of self-esteem and demographic information of age and gender, χ^2^ (104) = 200.57, *p* < 0.001, CFI = 0.94, NNFI = 0.92, SRMR = 0.06, RMSEA = 0.07 (see Figure 1). Life satisfaction at time 1 positively predicted life satisfaction at time 2, *b* = 0.43, β = 0.38, *p* < 0.001, indicating a significant autoregressive effect. More importantly, after controlling the autoregressive effect of life satisfaction at time 1 and the effects of self-esteem, reward for application at time 1 significantly predicted life satisfaction at time 2, *b* = 0.34, β = 0.17, *p* = 0.033. Reward for application at time 1 positively predicted reward for application at time 2, *b* = 0.32, β = 0.27, *p* = 0.003, while life satisfaction at time 1 failed to predict reward for application at time 2 with the control of reward for application at time 1 and self-esteem, *b* = 0.01, β = 0.02, *p* = 0.852.

#### 3.2.2. The Interplay between Reward for Application and Self-Esteem

Latent moderation analysis was performed to test whether the lagged effect of reward for application on life satisfaction was contingent on one’s self-esteem. With the LMS approach, a significant interaction effect between reward for application at time 1 and self-esteem was found on life satisfaction at time 2, *b* = −0.66, *p* = 0.044. This negative interaction effect indicated that the beneficial effect of reward for application on life satisfaction was enhanced by the decrease in self-esteem. Simple slope analysis also revealed that for those who had low self-esteem (one *SD* below mean value), the beneficial effect of reward for application on life satisfaction was strong, *b* = 0.63, *p* = 0.016, while the beneficial effect of reward for application was blocked among those who had high self-esteem (one *SD* above mean value). Finally, it is noteworthy that self-esteem did not moderate the link between life satisfaction at time 1 and reward for application at time 2, *b* = 0.07, *p* = 0.957.

## 4. Discussion

Conceptually, the effect of reward for application on life satisfaction is paradoxical. Either a positive, a negative, or a null effect can be expected. Due to some methodological limitations, previous investigations were not able to make a robust conclusion on the relationship between reward for application and life satisfaction. To address these limitations, we examined this relationship by (1) using a longitudinal approach to capture the temporal dynamics and to minimize the problem of common method variance, (2) including both samples of young adults and adolescents to expand the generalizability, and (3) controlling the confounds of self-views and examining the multiplicative effect between self-views and worldviews.

Across two cross-lagged panel studies, we found that reward for application had a positive lagged effect on life satisfaction on both samples of young adults (study 1) and adolescents (study 2). Importantly, this lagged effect was positive, indicating that reward for application did enhance one’s life satisfaction. This lagged effect was also over and above the potential confounds of self-views (namely, self-rated personality traits and self-esteem). In the reverse direction, life satisfaction was found to have no lagged effect on reward for application in both short (three months) and long (one year) time lags. Interestingly, we found support for the multiplicative effect between worldviews and self-views. Specifically, self-esteem had attenuated the positive effect of reward for application. Reward for application only positively affected life satisfaction among those who had low self-esteem, while it had no effect on life satisfaction among those who had high self-esteem. Taken together, these findings enriched our understanding of the association between reward for application and life satisfaction.

### 4.1. The Temporal Effect of Reward for Application on Life Satisfaction

A positive relationship between reward for application and life satisfaction has been consistently demonstrated in the literature [9,25]. However, these cross-sectional findings did not provide any insights on the direction of effects. In the present research, the findings from two longitudinal studies filled this gap. Reward for application had a positive effect on lagged life satisfaction, while the reverse direction was not supported.

As a belief that the exertion of effort will lead to positive outcomes, reward for application is considered to be a “good force” to drive positive outcomes. Thus, the beneficial effect of reward for application found in the present research is indeed consistent with its conceptualization. However, the mechanisms of how reward for application affects life satisfaction are unclear. People high in reward for application tend to exert more effort [22], and this may help them accomplish more. These accomplishments may serve as an underlying mechanism that channels the beneficial effect of reward for application to one’s life satisfaction [47]. Further investigations should be conducted since the empirical association between reward for application and accomplishments is inconclusive [20]. Moreover, it is possible that reward for application may equip people with more adaptive psychological resources, which in turn enhance one’s life satisfaction. For instance, reward for application is linked with being optimistic [10], having a chronic tendency to feel hopeful [48], and a proneness to feel grateful [49].

Nevertheless, this beneficial effect of reward for application observed in this research does not rule out the possibility of its dark side. It is methodologically possible for a larger positive effect to cancel out a weaker negative effect, resulting in a beneficial effect in observation. Reward for application can regulate daily behaviors, such as motivating one to work harder after a failure. The daily process of behaving effortfully and in a disciplined manner can be difficult and painful [50], but the successful progress or rewarding outcome at the end can help boost one’s life satisfaction. Since the present research focused on a relatively long-term consequence for reward for application (i.e., the lagged life satisfaction after three months/one year), the short-term effect (e.g., daily or momentary influences) of reward for application is still unclear. Future studies should be conducted using different research designs.

### 4.2. The Interplay between Reward for Application and Self-Esteem

Moving beyond the additive effects of worldviews and self-views in previous studies, the present research tested the multiplicative effect between worldviews and self-views. We found that the lagged effect of reward for application on life satisfaction was contingent on self-esteem. Particularly, reward for application only positively affected life satisfaction among those who had low self-esteem, but not among those with high self-esteem. These findings highlight an important role of reward for application in negative conditions. Interestingly, previous research revealed a similar pattern of results. Leung and Bond [14] found that GDP per capita was negatively associated with a belief in reward for application. They proposed that difficult and challenging situations may accentuate the importance of reward for application because exerting effort is likely to bring immediate and visible improvement in a bad situation [13].

Self-esteem refers to a positive evaluation of self [46]. People with low self-esteem hold a negative evaluation of the self and have a poor sense of self-regard, self-worth and self-acceptance. Therefore, low self-esteem may reflect a psychologically poor, difficult and challenging condition. As in previous research, it is possible that reward for application will become especially important in this negative condition. For instance, when the exertion of effort brings accomplishment, those who have poor self-worth are more likely to consider these accomplishments as substantial and important than those who have high self-esteem. People with low self-esteem often regard their performance as inadequate and expect to fall short of success, whereas those with high self-esteem expect to succeed [51]. Hence, the improvements and accomplishment brought by effortful behaviors are likely to be more visible and psychologically substantial to those with low self-esteem than those with high self-esteem. This may explain why the effect of reward for application on one’s life satisfaction is stronger among those who have a poor sense of self-worth.

### 4.3. Limitations and Future Directions

This study has some limitations. First, the present research was conducted in a collectivistic culture (i.e., Hong Kong). It has been proposed that in a collectivistic culture, the effects of worldviews would be stronger, and the effects of self-views would be weaker than in an individualistic culture [31]. It is possible that the optimistic worldview of reward for application cannot affect one’s life satisfaction over and above the effects of self-views in individualistic cultures because members of individualistic cultures put more emphasis on themselves instead of others and their social environment. Future studies can be conducted to examine the generalizability of the current findings on individualistic cultures.

As aforementioned, the present research focused on a relatively long-term consequence for reward for application (i.e., the lagged life satisfaction after three months/one year). This long-term focus fails to implicate the relatively shorter processes in reward for application. For instance, reward for application may regulate daily behaviors and impact momentary psychological well-being. To unveil different underlying processes across a broader set of time frames, future research can adopt experience sampling or diary sampling methods to investigate both long- and short-term effects of reward for application.

Finally, the present research found a moderation effect on self-esteem but not on self-rated personality traits. Conceptually, self-esteem is more domain-general than self-rated personality traits, and it fits the generalized expectancy in reward for application [14]. Methodologically, construct reliability also affects the likelihood of detecting a moderation effect [52]. In the present research, the reliability estimates in self-rated personality traits (average α = 0.63, ranging from 0.48 to 0.71) are lower than the reliability estimate in self-esteem (α = 0.80). Therefore, these two factors may confound the moderation effect observed in this research. Future studies should be conducted to include a more reliable measurement of personality traits as well as other domain-general self-views, such as self-efficacy.

## 5. Conclusions

The present research extends previous studies in worldviews by overcoming three methodological flaws, namely common method variance, failure to capture temporal dynamics, and not controlling self-views. Going beyond the additive effects in the literature, we also examine the multiplicative effect between worldviews (namely, reward for application) and self-views (namely, self-esteem and self-rated personality traits). With two samples of young adults and adolescents, we reveal a lagged effect of reward for application on life satisfaction after three months and one year. This lagged effect is also contingent on self-esteem, enriching our understanding of the effect of reward for application under different circumstances.

## Figures and Tables

**Table 1:** Descriptive statistics and bivariate correlations in studies 1 and 2.

	1	2	3	4
1. T1 RFA	3.69(0.59)/3.95(0.51)	0.24 **	0.24 **	0.23 **
2. T1 LS	0.29 ***	4.22(1.14)/4.68(1.03)	0.13 ^†^	0.44 ***
3. T2 RFA	0.55 ***	0.18 *	3.71(0.66)/3.54(0.58)	0.34 ***
4. T2 LS	0.32 ***	0.51 ***	0.35 ***	4.31(1.34)/4.27(1.10)

RFA = reward for application; LS = life satisfaction. Intercorrelations in study 1 are provided below the diagonal while those in study 2 are provided above the diagonal. Mean and standard deviation in study 1 (left) and study 2 (right) are provided on the diagonal. ^†^ *p* < 0.10, * *p* < 0.05, ** *p* < 0.01, *** *p* < 0.001.
